# Image-Based Medical Navigation Systems for Cardiac Interventions: Recent Technological Advances

**DOI:** 10.1007/s13239-026-00830-4

**Published:** 2026-03-30

**Authors:** Luís C. N. Barbosa, João L. Vilaça, Siobhán Moane, Pedro Morais

**Affiliations:** 1 2AI-Applied Artificial Intelligence Laboratory, School of Technology, IPCA, Barcelos, Portugal; 2 LASI-Associate Laboratory of Intelligent Systems, Guimarães, Portugal; 3 LIFE Biosciences Research Institute, TUS- Technological University of the Shannon: Midlands Midwest, Limerick, Ireland

**Keywords:** Review, Multi-modality imaging, Image tracking, Artificial intelligence, Cardiac catheterization procedures, Medical navigation systems

## Abstract

**Background:**

The application of catheter-based treatments for a growing range of structural heart diseases (SHD) has significantly increased over the past five years, driven by technological advances in medical navigation systems, particularly those based on medical imaging. Multimodal cardiac imaging plays a crucial role in these systems by combining complementary anatomical, morphological, and functional information, thereby increasing diagnostic accuracy and improving the effectiveness of cardiovascular interventions and clinical outcomes. However, multimodal imaging poses challenges, including intermodality misalignment and the need to determine optimal integration methods for data from different imaging modalities.

**Methods:**

This article reviews the state-of-the-art image-based medical navigation systems used in catheter-based cardiac procedures. The review covers the period from 2019 to 2023 and includes a total of 44 articles. The methodologies in these studies are grouped into six main categories: Image Enhancement and Tracking, Image Fusion and Reconstruction, 3D Modeling/Printing, Extended Reality, and Artificial Intelligence (AI).

**Results:**

Most studies involve multimodality imaging, combining or transferring information across different modalities. The review emphasizes that current multimodal imaging techniques enhance the accuracy of procedures such as left atrial appendage closure (LAAC), transcatheter aortic valve implantation (TAVI), and catheter ablation therapy. These techniques rely on combinations of imaging modalities such as fluoroscopy, ultrasound, and computed tomography (CT) to enable real-time guidance and precise navigation during minimally invasive interventions.

**Conclusion:**

By integrating data from multiple sources, these systems improve diagnostic reliability and procedural success, meeting the complex demands of SHD treatment. Despite advances, real-time surgical guidance remains a major challenge, underscoring the need for continued research in this area.

## Introduction

Currently, it is estimated that millions of endovascular catheters are inserted in humans annually throughout the world. This number can be attributed to the constant increase in minimally invasive transcatheter cardiovascular intervention (TCI) procedures developed/introduced in recent years [1]. This growth in transcatheter cardiovascular interventions has been largely driven by the aim of enhancing patient comfort, minimizing pain and scarring, reducing the risk of infection, and shortening the recovery time compared to traditional open-heart surgery [1–3].

Several TCI procedures are commonly performed using endovascular catheterization. These include Percutaneous Coronary Intervention (PCI) [4], Left Atrial Appendage Closure (LAAC) [5], Transcatheter Aortic Valve Implantation (TAVI) [6], Mitral Valve Replacement (MVR) [3], Atrial Septal Defect Closure (ASDC) [7], radiofrequency current ablative cardiac catheterization and Endovascular Aneurysm Repair (EVAR) [8], among others. Each of these procedures have a high effectiveness rate, however limitations and complications are still found [1].

In response to the increasing complexity of transcatheter interventions, the demand for improved guidance and navigation systems is constantly increasing [9]. In this context, cardiac imaging techniques can provide anatomical and functional information about the heart muscle, assisting medical experts in the aforementioned procedures. Collectively, numerous different imaging modalities play a key role in providing all the data needed in these various procedures, namely, to define the optimal timing, the most suitable approach and to prevent potential complications during the intervention [10]. Commonly used imaging modalities include X-ray (or fluoroscopy), Computed Tomography (CT), Magnetic Resonance Imaging (MRI), and Echocardiography (Echo).

**Fig. 1:**
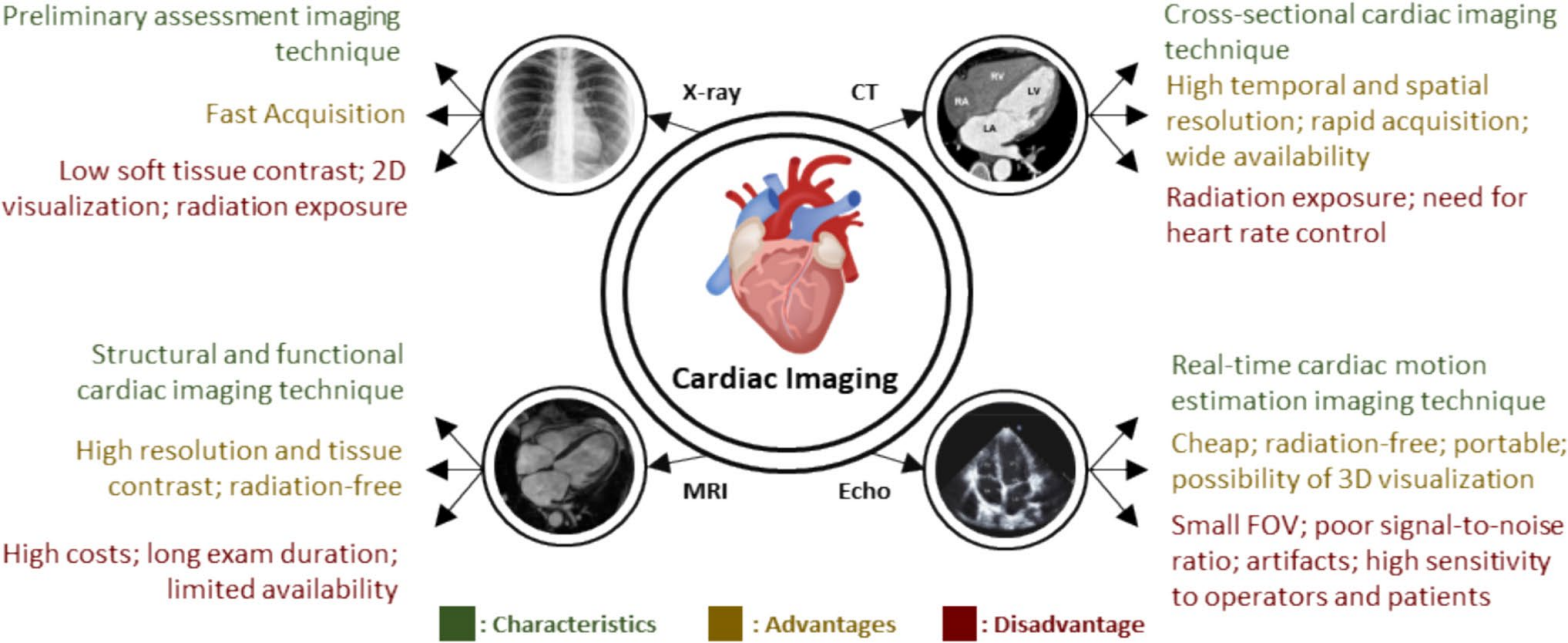
Examples of cardiac imaging techniques from different modalities with their characteristics, advantages, and disadvantages [15]. Here, X-ray, CT, MRI and Echo images adapted from [50] [54] [55] [56], respectively

As shown in Figure 1, each imaging modality conveys specific information. In detail, the fluoroscopy is mandatory during TCI, where its great advantage is its rapid acquisition and large field of view (FOV), however it presents low contrast in soft tissues, only allowing 2D visualization, and exposes the patient to radiation [11]. In relation to the CT image, it is a cross-sectional cardiac imaging technique, which presents high spatial resolution, rapid acquisition, and wide availability. However, it emits radiation to the patient and its application during TCI is unfeasible due to logistical reasons in most of the world [12]. MRI imaging is a structural and functional cardiac imaging technique which presents high resolution and tissue contrast, which is free of radiation for the patient [11]. However, it has a high cost, is an examination procedure which takes a long time and has limited availability. Lastly, echo imaging is a real-time cardiac motion estimation imaging technique. It is a low-cost, radiation-free, portable imaging technique, with the possibility of 2D or 3D visualization. However, it has a small FOV, poor signal-to-noise ratio, presents artifacts and has high sensitivity to operators and patients [13, 14].

At times, a single modality may provide insufficient and/or ambiguous information regarding the condition of the heart, which is a non-rigid and dynamic structure [15]. Recently, several studies have indicated that combining complementary information from multimodal cardiac images can be important for establishing a more accurate diagnosis or assisting physicians in catheter manipulation during cardiac interventions [10, 14]. Cardiac image fusion (IF) is thus a widely adopted strategy to guide transcatheter cardiac interventions. These techniques mainly focus on combining fluoroscopic imaging with other modalities, such as CT, Transoesophageal Echocardiogram (TEE), Transthoracic Echocardiogram (TTE) or MRI [16].

In this regard, the general objective of this study is to review the current state of the art of image-based medical navigation systems in catheter-based cardiac procedures. Several recent reviews have addressed specific aspects of this field, including modality-focused reviews centered on fluoroscopy, echocardiography, or MRI, as well as surveys primarily dedicated to artificial intelligence techniques for image segmentation, tracking, or decision support. In parallel, a substantial number of clinically oriented reviews focus predominantly on in-hospital validation, procedural outcomes, and safety of individual navigation technologies.

In contrast, the present review adopts a system-level perspective on image-based medical navigation for catheter-based cardiac interventions. Rather than assuming joint implementation of multiple technologies, the proposed categorization organizes heterogeneous and often modality-specific contributions according to their functional role within a common navigation pipeline. This approach enables a unified analysis of clinical advances alongside emerging technical solutions, highlighting how image enhancement, tracking, fusion, 3D modeling, extended reality, and artificial intelligence address complementary stages of navigation workflows. By explicitly identifying current fragmentation, integration gaps, and convergence opportunities, this review aims to bridge clinical practice with technological innovation and to provide insights into future directions for integrated navigation systems.

The review is organized into sections. Section “Review Methodology” details the research methodology adopted while undertaking the review of the literature. Section "3D Model/Printing" summarizes the imaging modalities widely used in cardiology. Section “Discussion” details the results of the articles found, organized by categories. Section “General current state of research and perspectives” discusses the results found. Section 6 provides an analysis of the current research and future perspectives. Finally, section 7 provides the final conclusions of the study developed.

## Review Methodology

In this section, the methodology used for conducting the present review is described. A computer-assisted search was performed by the first author in the Web of Science, and PUBMED databases using combinations of the following keywords as described in (Equation 1).

**Fig. 2:**
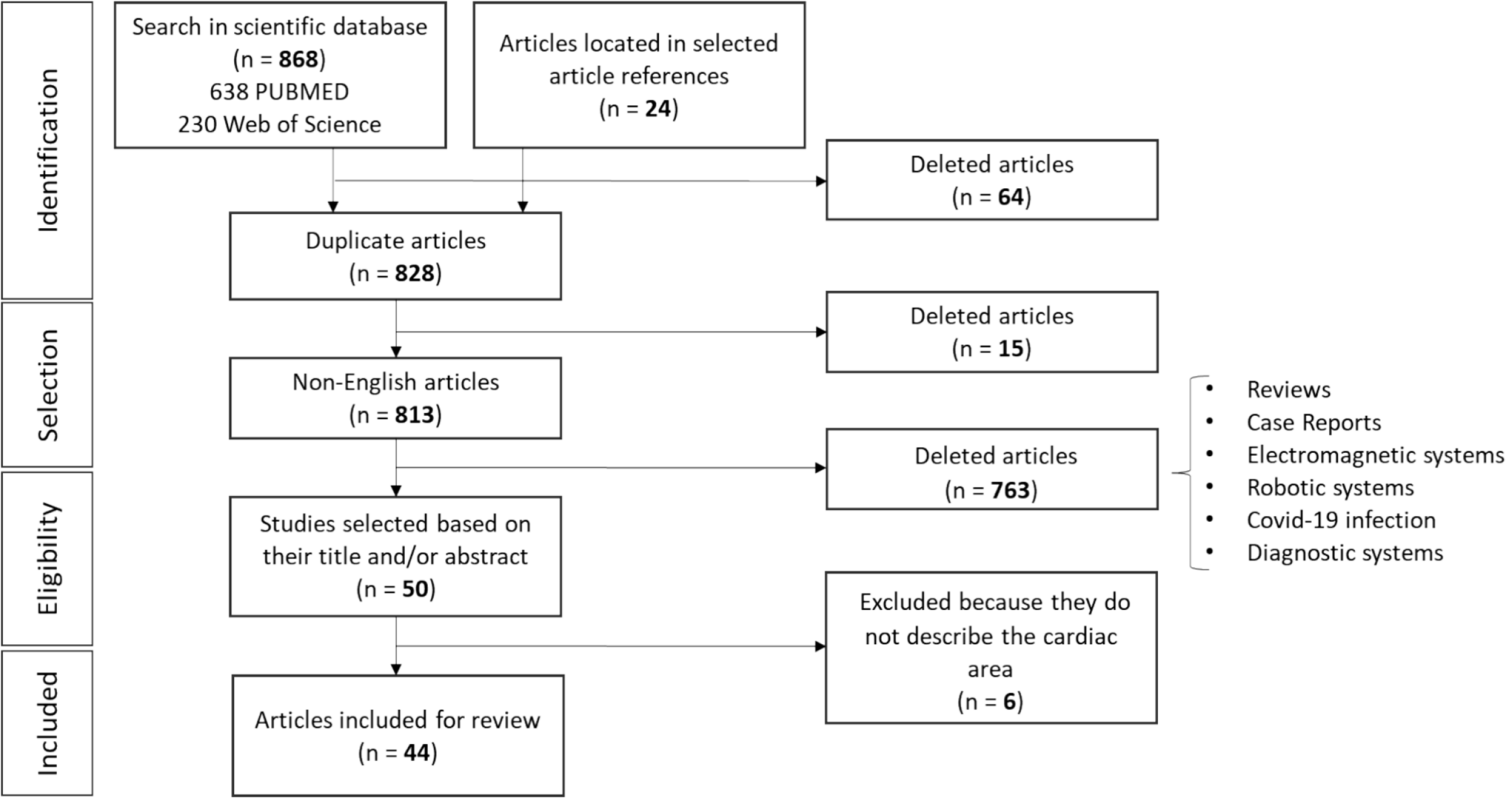
Bibliographic research results flow chart

The research covered the period from 2019 to 2023 (5 years), totaling 868 articles. This time window was selected to capture the most recent technological advances in image-based medical navigation systems, particularly the rapid methodological development and increasing research interest in artificial intelligence and machine learning techniques applied to image-based navigation [17] [18]. Earlier foundational developments up to 2019 have been extensively reviewed in the literature, including comprehensive reviews on multimodal imaging, image fusion, and navigation frameworks [19] (Nobre et al., 2020b). Rather than duplicating these efforts, the present review focuses on subsequent advances. The upper limit of 2023 corresponds to the most recent complete publication period at the time of the literature search, allowing sufficient time for consolidation and critical assessment of emerging techniques. In addition, a five-year window is commonly adopted in recent review studies to provide a current yet comprehensive overview of rapidly evolving fields (Nobre et al., 2020b). Additionally, 24 articles were identified in the references of previously known studies (Figure 2). As the searches were carried out in two databases, following the same methodology (Equation 1), the first step was to identify duplicate studies. In this sense, 64 duplicated articles were excluded. Subsequently, 15 non-English articles were excluded. After the selection stage, a total of 813 articles were analysed based on their title and abstract, to select only those that fit the scope of the review. Following the eligibility analysis, 763 studies were excluded, representing, for example: reviews, non-image-based techniques (e.g. electromagnetic/robotic systems), studies dedicated to Covid-19 infection, diagnostic systems, among other topics. These exclusion criteria were applied to ensure that the selected studies were directly aligned with the thematic subtopics defined in this review and with the scope of image-based navigation systems for cardiac interventions. This resulted in a total of 50 articles eligible for full-text analysis. After thoroughly reading each article, 6 were excluded from the review due to their lack of focus on cardiac studies, resulting in a total of 44 articles included, reviewed, and described in this review. It is important to note that the entire selection and analysis process of the articles identified in the review was carried out using the Rayyan software (https://www.rayyan.ai/).

## Results

**Fig. 3:**
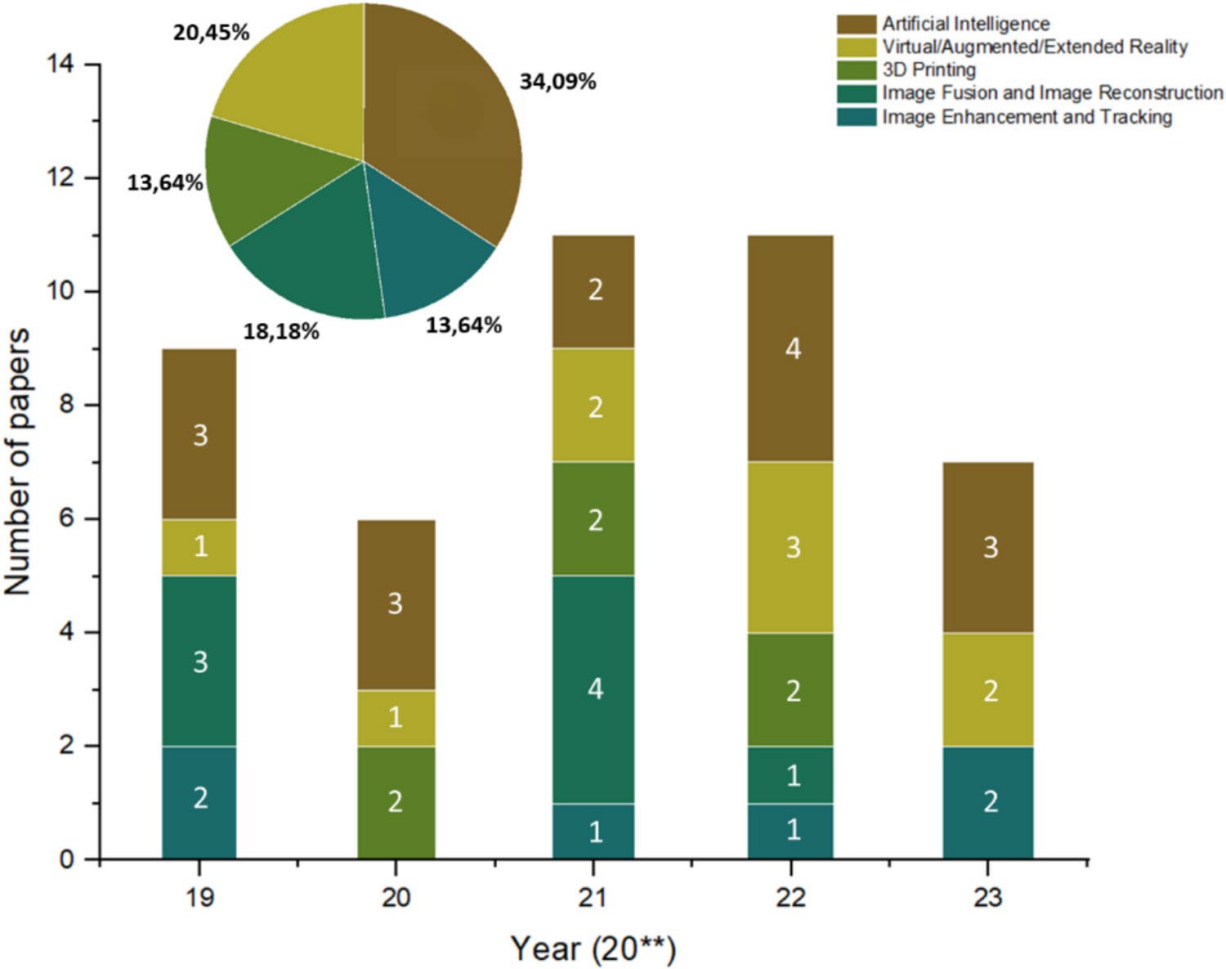
The distribution of reviewed articles on image-based cardiac medical navigation systems by year and task

A decision was made to organize the articles selected for review into categories according to the main area of research proposed. The selected areas were: (i) Image Enhancement and Tracking; (ii) Image Fusion and Image Reconstruction; (iii) 3D Model/Printing; (iv) Virtual/Augmented/Extended Reality; and (v) Artificial Intelligence. Each of them was analysed individually, and the respective articles associated with each category were then organized by year. Additionally, the representative percentage distribution of each category was observed (Figure 3).

### Image Enhancement and Tracking

To guide the reader, the authors note that although image quality enhancement and image-based tracking pursue distinct technical objectives, they are closely interrelated within image-based navigation workflows. For this reason, they are discussed jointly in this section, reflecting their complementary roles in enabling real-time catheter guidance.

###  Image quality enhancement and enhanced visualization.

Guidewires play a crucial role as a foundational tool in the practices of radiologists and interventional cardiologists. They are used to navigate through the vasculature, facilitating the advancement of larger and less maneuverable devices, such as catheters, along the wire to the target vessel. This process enables quick access to the desired areas of the vascular system. During cardiac catheterization procedures (CCP), the real-time tracking and precise visualization of instruments, such as guidewires and selective or steerable catheters, are critical for the success of endovascular interventions.

However, significant technical challenges arise when implementing these interventions in MRI environments. One of the primary challenges is the limitation on the use of ferromagnetic materials due to the strong magnetic field, which prevents the use of traditional materials in catheters and guidewires. Additionally, polymeric materials, which are often used for their safety in MRI environments, have low magnetic susceptibility, making their real-time visualization and localization during procedures challenging. This gap has motivated the development of new image enhancement and tracking techniques that integrate various imaging modalities and advanced image processing algorithms.

###  Image-based tracking for navigation (guidewires and catheters).

**Fig. 4:**
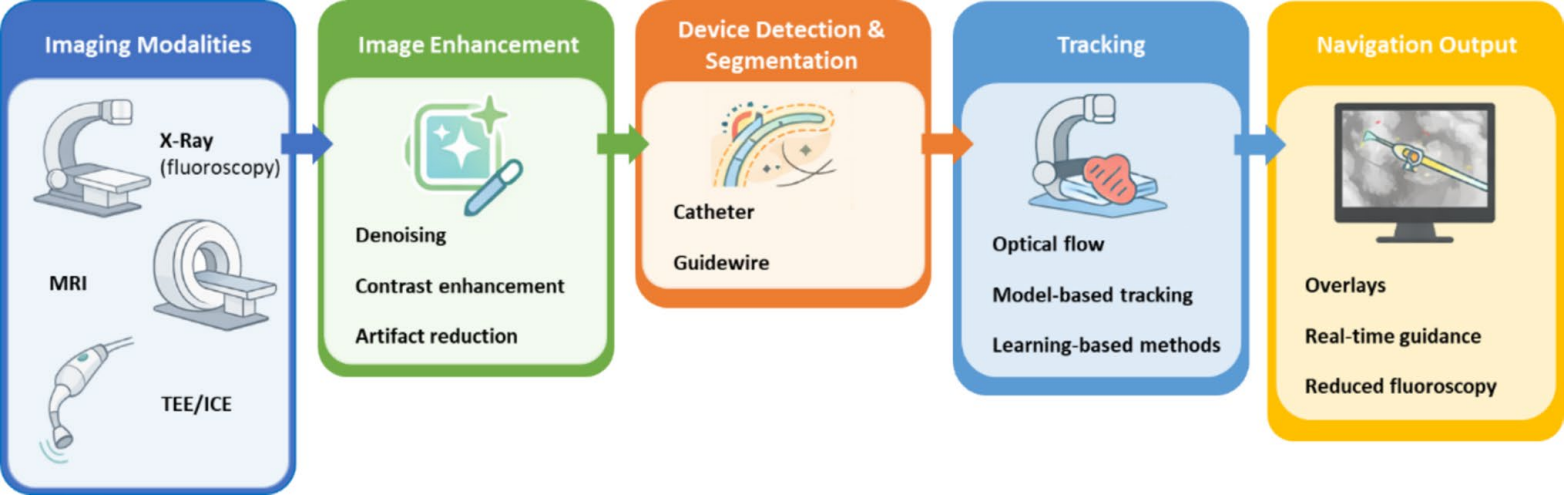
Conceptual overview of image enhancement and tracking in image-based cardiac navigation. The figure summarizes a typical workflow in which multimodal imaging data (e.g., X-ray fluoroscopy, MRI, and TEE/ICE) are processed through image enhancement and device tracking stages to support real-time catheter navigation. Image enhancement improves visibility and contrast, enabling robust detection and tracking of catheters and guidewires using learning-based methods. The figure provides a high-level synthesis of the approaches discussed in Section 3.1

The methodologies for real-time catheter tracking in minimally invasive intervention environments are largely based on image processing techniques. One of the most common methods is the use of optical flow, which calculates pixel movement between successive frames of an image sequence, allowing for high-precision catheter tracking [20]. This technique is particularly useful in situations where the catheter is in constant motion or where the field of view changes rapidly. In addition to optical flow, other image processing techniques, such as edge detection [21] and model-based tracking [20], have been applied to improve device visualization. These techniques allow the automatic identification of the shape and position of the catheter in the acquired images, even in low-contrast scenarios or when interference from surrounding anatomical tissues is present. Table 1 shows a summary of the scope of works reviewed, considering the topic, Image Enhancement and Tracking. Figure 4. illustrates a conceptual pipeline in which multimodal imaging sources (X-ray, MRI, and TEE/ICE) are combined with image enhancement and tracking strategies to support real-time catheter navigation [22] propose an innovative method for automatic guidewire segmentation and tracking during robotic intravascular catheterization. Using X-ray, the study developed a multiscale enhancement filtering technique for pixel detection and vassalless? measurement, paired with morphological operations to ensure guidewire pixel connectivity. Validated in 12 angiographic sequences from in vivo trials on rabbits, the system achieved a segmentation accuracy of 99.5% and tracking errors as low as 1.938 mm. This approach enhances robotic catheter navigation for PCIs, reducing operational hazards and improving tool visualization [23] proposed a method based on MRI for real-time automatic catheter tracking using MRI image slices. Their approach uses image processing algorithms based on volumetric reconstruction techniques and sequential slice alignment to monitor catheter movement in a three-dimensional environment. The system was designed for applications in CCP, demonstrating an effective proof of concept for continuous catheter tracking in minimally invasive procedures [24] developed a multimodal catheter tracking system using X-rays and MRI. Their methodology employs IF to combine the high spatial resolution of X-rays with the soft tissue characterization provided by MRI. Catheter tracking is performed by detecting contrast changes in X-ray and MRI images, which are correlated in real time using feature matching algorithms [25] focused on the combined use of X-rays and Intracardiac Echocardiography (ICE) to track catheters during cardiac ablation procedures. Instead of traditional image registration between the 2D ICE and X-ray images, they implemented a low-fluoroscopy workflow that relied heavily on real-time ICE visualization and electroanatomic (EA) mapping systems to track catheter position. This allowed for the construction of a 3D anatomical shell of the atrium, effectively aligning spatial information from ICE within the EA map, thus ensuring accurate catheter navigation and reducing dependence on fluoroscopy [26] applied a novel fibreoptic ultrasound sensor (FOUS) integrated into a steerable catheter to enable real-time tracking during TEE-guided cardiac procedures. Unlike traditional tracking systems, this method uses a passive receiver, the FOUS, which detects ultrasound pulses emitted by the TEE probe and estimates the catheter tip's position based on time-of-flight measurements. A key innovation was the use of bend-insensitive optical fibre, which maintained high sensitivity even under tight catheter deflections (down to a bend radius of 3 mm), ensuring robust tracking performance regardless of anatomical complexity. Finally, [27] described a technique to guide the Transseptal puncture (TSP) procedure using ICE. They utilized anatomical feature detection algorithms that identified reference points on the interatrial septum to guide catheter positioning. This approach enabled more accurate navigation during the TSP procedure, reducing operator dependency and enhancing procedural safety. Moreover, the authors demonstrated that automatic detection of anatomical landmarks in ICE images can be integrated in real time into navigation systems, representing a significant step forward in the automation and reliability of intraprocedural guidance.

**Table 1:** Summary of related representative review works related to image enhancement and tracking

Study	Modality	Goal	Method	Application
[22]	X-Ray	Guidewire segmentation and tracking.	Multiscale enhancement filtering for pixel and vesselness detection.	Robotic catheterization.
[45]	MRI	Real-time catheter tracking.	Saturated slices + real-time processing.	CCP
[24])	X-Ray & MRI	Catheter/wire detection.	Tensor-based method, motion compensation, 2D–3D registration.	CCP
[25]	X-Ray & ICE	Catheter guidance to right atrium, double TSP.	Fluoroscopy + intracardiac ultrasound.	CCP
[27]	ICE	TSP technique using ICE.	Sheath + Brockenbrough needle placed at fossa ovalis.	TSP
[26]	TEE	Catheter tracking strategy.	Ultrasound-based, curvature-insensitive tracking.	CCP

*LAAO* left atrial appendage occlusion

In practice, image enhancement techniques directly support tracking performance by improving device visibility, contrast, and anatomical context, particularly in low-contrast or multimodal imaging environments.

While advances in image enhancement and tracking techniques have allowed for greater accuracy in catheter-guided procedures, some challenges remain. One key issue is the real-time synchronization of data streams from multiple imaging modalities with inherently different acquisition rates and resolutions. Studies such as [28] addressed these issues by employing machine learning-based registration algorithms and anatomical landmark detection to fuse 3D echocardiography with X-ray fluoroscopy in real time. Similarly, [29] [30] highlighted the difficulties in fusing high-resolution CT or MRI with lower-resolution fluoroscopy, resolving these by using phase-matched cardiac models and spatial calibration. These approaches reflect the broader need for robust intermodality fusion techniques that can handle resolution mismatches and temporal misalignments during minimally invasive cardiovascular interventions.

### Image Fusion and Image Reconstruction

Although image reconstruction and image fusion frequently address different stages of the image processing pipeline, they are tightly coupled in image-based navigation systems. In this review, reconstruction and fusion are discussed together to provide an integrated perspective.

Table 2 reports the main studies found related to the topic of image fusion and image reconstruction. It is divided into two large groups, which include the Software group that is dedicated to technological studies, and the group of studies with application to a larger population, with a considerable number of patients. Complex transcatheter interventions often require 3D soft tissue imaging guidance. Sometimes, a single imaging modality cannot achieve a 3D visualization of cardiac structures. In this sense, different authors argue that fusion imaging (FI) with live X-ray fluoroscopy can potentially improve and simplify procedures and achieve 3D visualization [28].

**Table 2:** Summary of related representative review works related to IF

	Author	Modality	Goal	Population	Main conclusion
Software	[9]	X-Ray & MRI & CT	Software for IF, navigation, and point documentation in transcatheter interventions.	N/A	Qt-based system enabling cross-modal registration and fusion using VTK.
Application in a Population	[30]	X-Ray & MRI	Evaluate benefits of XFM in congenital heart disease interventions.	46 patients.	XFM offers 3D soft tissue guidance, reduces fluoroscopy time.
	[31]	X-Ray & CT	Fusion of angiography and SPECT-MPI for PCI guidance.	30 patients.	Accurate and feasible fusion improves decision-making in PCI.
	[29]	X-Ray & CT	Evaluate fusion of CT and XR for LAAO guidance.	82 patients	Reduces procedure time, radiation, and contrast use.
	[58]	X-Ray & CT	Assess reproducibility of fusion in aortic annulus for TAVI.	50 patients.	Potential for safer TAVI via integrated MDCT fusion.
	[59]	X-Ray & US	Evaluate co-registration of IVUS and angiography for PCI.	3 phantom models & 25 patients.	System effective in both phantom and human images.
	[28]	X-Ray & TEE	IF framework via TEE probe pose detection in real-time.	10 patient datasets.	Achieved 2.6 mm error—clinically acceptable.
	[57]	X-Ray & TEE	Assess IF for LAAC procedures.	155 patients	Reduced total and puncture time, and contrast usage.

*MDCT* reconstruction of multidetector CT, *EP* electrophysiology procedures, *XFM* X-ray fused with MRI

In this context, image reconstruction primarily refers to the generation of three-dimensional anatomical models from pre- or intra-procedural imaging, whereas image fusion focuses on the integration of these reconstructed models with live imaging modalities such as fluoroscopy, MRI, or ultrasound to support real-time navigation. Although image fusion is conceptually related to image registration, it is discussed here from the perspective of multimodal information integration rather than geometric alignment alone.

[9] proposed a software system for IF, real-time navigation, and working point documentation during transcatheter interventions performed under X-ray guidance. The system works by using algorithms that integrate fluoroscopic X-ray images with 3D anatomical models derived from CT or MRI. The IF is achieved through a rigid registration process, where corresponding reference points in the 3D images (CT or MRI) and 2D projections (X-ray) are identified and aligned. This creates an accurate correspondence between the two modalities, allowing the 3D anatomical model to be overlaid directly onto the X-ray images in real time. The software employs advanced projection geometry techniques to calculate the necessary transformations between the coordinate systems of the different imaging modalities. It also implements motion compensation and catheter tracking algorithms, enabling the dynamic adjustment of the image overlay in response to patient movements (such as heartbeats and respiration). During the interventions, physicians can manipulate the images in real time, navigating medical devices with greater precision, relying on the 3D visualization of cardiac structures projected onto the 2D X-ray image. This methodology not only improves procedural accuracy but also has the potential to reduce radiation exposure and procedure time, as physicians gain a clearer view of the relevant cardiac structures and medical devices during the intervention.

[28] propose a new framework for IF by detecting the position of the TEE probe in real-time X-ray images. The framework does not require any manual initialization. Instead, it uses a cascade classifier to calculate the position and rotation angle in the plane of the TEE probe. The remaining degrees of freedom are determined by rapidly marching against a library of models. The proposed framework is validated on phantom and patient data. The study showed an average registration error of 2.6 mm, which is within typical clinical requirements.

[31] developed an innovative IF approach to enhance decision-making in PCIs. The method integrates anatomical data from fluoroscopic angiography (FA) with functional information from single-photon emission CT myocardial perfusion imaging (SPECT-MPI), creating accurate 3D representations of coronary arteries and the left ventricular epicardial surface. Fusion is achieved through deformable registration algorithms, which align anatomical and functional structures with high precision, even in the presence of challenges such as cardiac motion. In clinical tests with 30 patients, the method achieved average fusion errors of 3.84 ± 3.15 mm for left coronary arteries and 5.55 ± 3.64 mm for right coronary arteries, validating its clinical feasibility. This approach represents a significant advancement, enabling better correlation between anatomical stenoses and perfusion defects, with the potential to optimize revascularization outcomes.

[29] evaluated the use of 3D cardiac CT and fluoroscopy fusion for LAAO in 82 patients (41 in the fusion group and 41 in the control group). The study showed that the fusion group had a significant reduction in procedure time (44.73 ± 20.03 min vs. 63.73 ± 26.10 min), radiation dose (448.80 ± 556.35 mGy vs. 798.42 ± 616.34 mGy), and contrast volume (67.32 ± 18.65 ml vs. 90.98 ± 25.03 ml) compared to the control group. The approach was safe, with no significant differences in complications, highlighting its potential to improve procedural efficiency and safety. The original study reports radiation dose and contrast volume associated with the interventional procedure; however, it does not explicitly specify whether the contribution of the pre-procedural CT acquisition was included in these measurements.

**Fig. 5:**
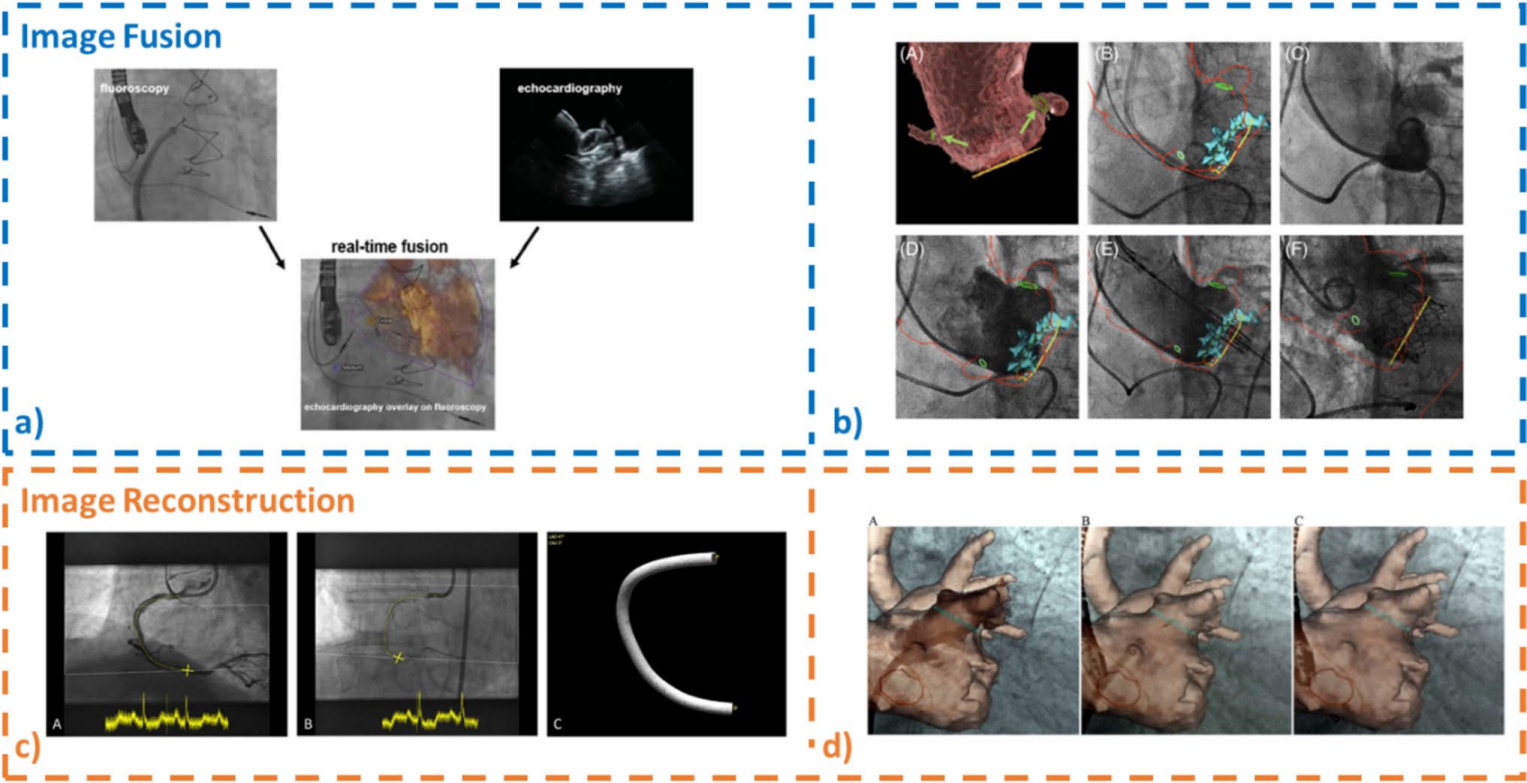
Representative examples of image fusion and image reconstruction strategies used in image-based navigation for catheter-based cardiac interventions. a) Real-time fusion of fluoroscopy and echocardiography to support complex structural heart interventions, enabling simultaneous visualization of devices and soft-tissue anatomy during procedures such as left atrial appendage closure. b) Multimodal fusion for transcatheter aortic valve implantation (TAVI), where pre-procedural CT-derived anatomical models, including the aortic root, coronary ostia, and calcifications, are overlaid onto live fluoroscopy to guide valve positioning and deployment. c) Example of image reconstruction for navigation, illustrating the three-dimensional reconstruction of an intravascular ultrasound (IVUS) catheter path from two angiographic projections acquired at different angles. d) Integration of reconstructed three-dimensional anatomical information with real-time fluoroscopy to support device deployment and verification of final positioning during structural heart interventions. Images reproduced with permission from [57–59] [29]

Figure 5 provides representative examples of both image fusion and image reconstruction approaches used in catheter-based cardiac interventions. Together, these examples highlight how image fusion and image reconstruction address complementary stages of the navigation pipeline, enabling enhanced anatomical understanding, improved device guidance, and more informed intra-procedural decision-making.

### 3D Model/Printing

**Fig. 6:**
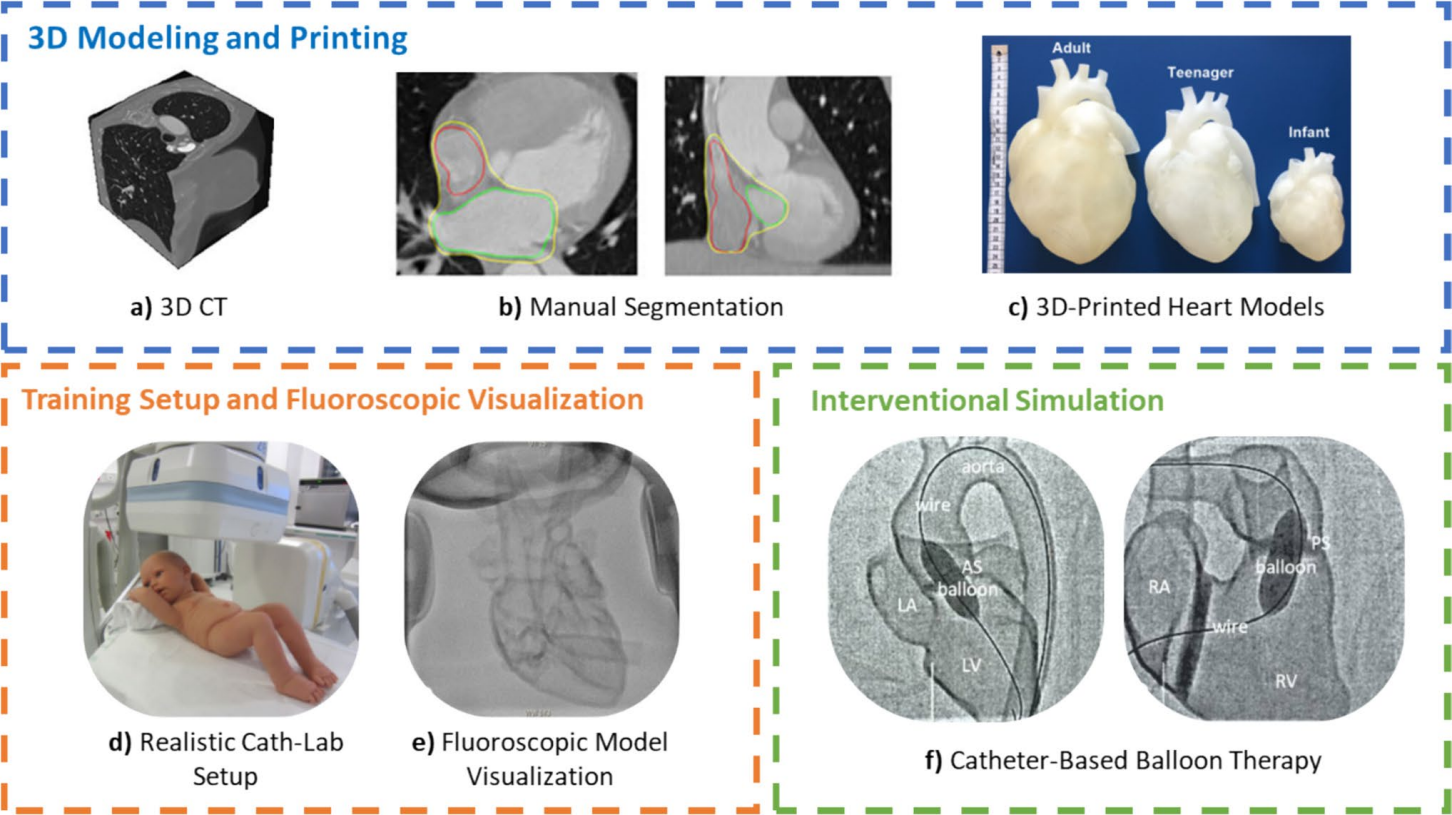
3D modeling, printing, and interventional simulation using patient-specific cardiac phantoms.The top panel illustrates the pipeline from volumetric cardiac CT acquisition and manual anatomical segmentation to the generation of patient-specific 3D-printed heart models at different scales (adult, teenager, and infant). The lower left panel shows the training setup, where a 3D-printed cardiac model is positioned in a realistic catheterization laboratory environment and visualized under fluoroscopy. The lower right panel presents fluoroscopic documentation of catheter-based balloon interventions performed within the 3D-printed heart model, illustrating its use for hands-on training and interventional simulation. *Images *[33]* reproduced under the terms of the Creative Commons Attribution 4.0 License (CC BY 4.0)*

This section addresses the topic of 3D models/printing, where \* MERGEFORMAT Table 3 summarizes the works found, related to the topic, detailing their contributions in this area. It assumes that catheter-based interventions are standard treatment options for cardiovascular pathologies. In this sense, patient-specific models could help train clinician skills as well as improve the planning of interventional procedures. Figure 6 illustrates the complete workflow for patient-specific cardiac 3D modeling and printing, starting from volumetric cardiac CT imaging and manual anatomical segmentation, followed by the generation of digital 3D heart models and their fabrication as physical cardiac phantoms using 3D printing technologies. The figure also highlights the integration of these phantoms into realistic training setups and fluoroscopy-guided interventional simulations.

**Table 3:** Summary of related representative review works related to 3D model/printing

Study	Modality	Goal	Population	Comments/main conclusion
[32]	X-Ray & CT & MRI & US	Develop patient-specific 3D-printed models for cardiovascular interventions.	N/A	High-resolution models (~30 µm) with material mimicking physiological biomechanics; tested with various catheterization techniques.
[33]	X-Ray & CT	Create a training module using 3D-printed hearts for pediatric cardiologists.	N/A	Enables basic and advanced CHD training with realistic diagnostic and interventional simulations.
[60]	X-Ray & TEE	Design and validate a physical TSP simulator using additive manufacturing.	14 experts	Reported as highly realistic in usability, haptics, and imaging fidelity.
[61]	X-Ray & TTE	Design of a TTE transducer holder for ASD/VSD closures.	23 patients	Allows real-time TTE monitoring during device closure.
[62]	ICE	Assess Cartosoundfam’s feasibility to reconstruct 3D LA anatomy from 2D ICE.	16 patients	Automatically identified LA structures with 69\% anatomical accuracy.
[34]	X-Ray & CT	Evaluate if CT-derived viewing angles improve LAA measurements.	28 reconstructions	Personalized CT views may enhance LAA size accuracy for Watchman device planning.

*ASD* atrial septal defect, *VSD* ventricular septal defect, *LA* left atrium, *CHD* congenital heart defects

[32], developed patient-specific flexible 3D cardiovascular phantoms, using CT imaging to serve as a training tool for cardiac interventionalists to plan the patient's cardiac catheterization intervention. The phantoms, after being constructed, were evaluated to assess whether the image models commonly acquired in this type of interventions, such as CT, MRI, X-Ray, and US can acquire quality images to help guide the surgical intervention.

Similarly, [33], printed 3D digital cardiac models, with or without heart defects, using the *Agilista 3200W printer (Keyence Corp, Osaka, Osaka, Japan)* and flexible silicone rubber material visible under X-rays. The authors demonstrated that it is possible to train several stages of a diagnostic procedure and intervention, as well as to practice and repeat them independently, which significantly reduced the fluoroscopy time. In the pre-surgical planning of a coronary intervention, it is crucial to accurately size the target cardiac structure, highlighting the importance of a thorough and careful process to increase the chances of successful intervention.

[34] acquired CT and fluoroscopy images to size the LAA, and subsequently performed the occlusion procedure using the Watchman™ device (Boston Scientific Corporation, MA, USA). In this study, 3D-printed patient-specific phantoms were created based on preprocedural CT data to simulate the left atrial anatomy and guide optimal C-arm angulations. The use of these phantoms allowed for a personalized assessment of fluoroscopic viewing angles, which, when referenced in-procedurally alongside 3D CT reconstructions, may improve the accuracy of landing zone diameter and length measurements for Watchman device deployment.

### Virtual/Augmented/Extended Reality

**Fig. 7:**
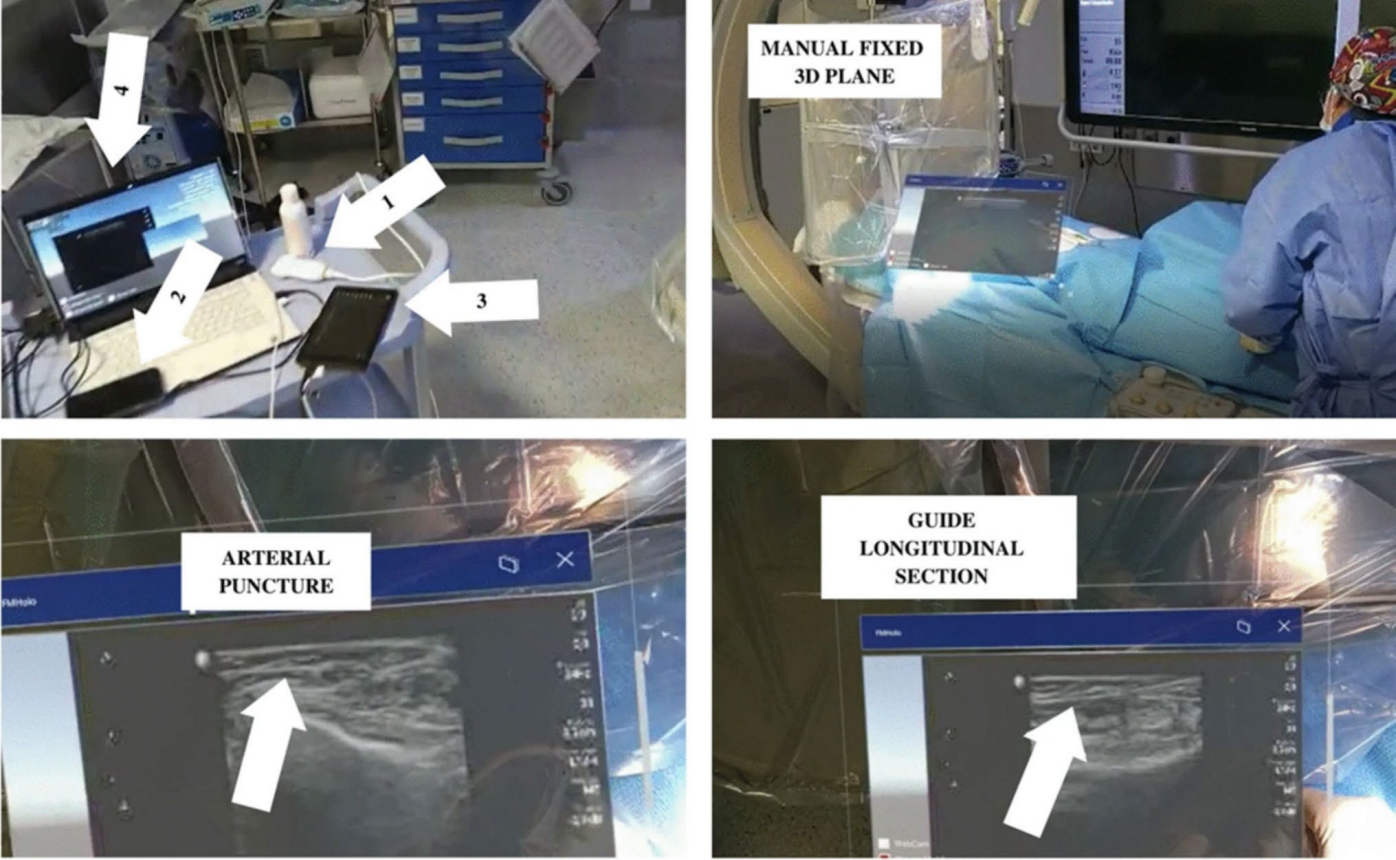
Example of extended reality assistance in interventional cardiology procedures. The figure illustrates a XR system used to support ultrasound guided vascular access in a clinical interventional setting, including hardware setup, real time image visualization, and augmented procedural guidance. Although not focused on intracardiac navigation, this example highlights how extended reality technologies can enhance procedural awareness, operator interaction with imaging data, and real time decision support, serving as a practical proof of concept for future XR based navigation systems in cardiac interventions. Reproduced from [43] under the Creative Commons Attribution 4.0 International License (CC BY 4.0)

Extended Reality (XR) represents any technology that combines computational environments with the real world, encompassing virtual reality, augmented reality, mixed reality, and other holographic displays. These techniques typically utilize head-mounted displays (HMDs) and body tracking technology, enabling 3D human-computer interactions where the virtual environment responds to user gestures or controllers. \* MERGEFORMAT Table 4 presents the most relevant studies in this field, exploring imaging modalities, methodologies, and key contributions. Figure 7 illustrates representative extended reality (XR) workflows applicable to image-guided cardiac and cardiovascular interventions, highlighting how augmented and mixed reality technologies can support procedural guidance and operator interaction with imaging data. The figure depicts XR-based applications including augmented ultrasound-guided vascular access, intraoperative manipulation and visualization of imaging information within the procedural environment, and remote mentoring scenarios enabling shared visualization and expert guidance. Although the example focuses on vascular access rather than intracardiac navigation, it demonstrates the potential of XR technologies to enhance spatial understanding, real-time decision support, and collaborative workflows, serving as a practical proof of concept for future XR-based navigation systems in catheter-based cardiac interventions.

**Table 4:** Summary of related representative review works related to extended reality

Study	Modality	Goal	Method	Application
[35]	CT	Develop a hybrid simulator using MR and 3D printing.	3D-printed cardiac phantom from CT integrated with mixed reality.	TSP and structural heart procedure training.
[36]	CT	Pre-procedural LAAO planning.	MR system for 3D holographic visualization from CT.	LAA morphology analysis for LAAO.
[37]	MRI	Real-time MR guidance using MR headset.	Subsampled MRI with GPU reconstruction visualized via HoloLens.	Accurate, low-latency system for procedure guidance.
[39]	CT & X-Ray	AR guidance for transcatheter procedures.	HoloLens displays catheter in 3D using CT/fluoroscopy fusion.	Planning, guidance, and training in interventions.
[40]	X-Ray & US	Evaluate MR headset use in cath lab.	Combines fluoroscopy, ultrasound, and data in headset view.	Streamlines workflow by removing need for multiple monitors.
[41]	X-Ray	Test MantUS™ for real-time US and needle tracking.	MR interface used in femoral/head-neck training models.	Improves vascular access precision and training.
[42]	CT & US	MR use in interventional cardiology (TAVI).	Real-time holographic anatomy display enhances ergonomics.	Better visualization and efficiency in vascular access.
[43]	US	MR-guided femoral puncture with US.	Image transfer and 3D display via HoloLens 2.	Real-time procedural support using MR.
[63]	MRI	Assess AR impact in virtual VT ablation.	2 virtual hearts used for navigation accuracy tests.	AR improves catheter targeting and ablation outcomes.

[35] proposed integrating a 3D visualization system into training platforms for transcatheter interventions. The system combines a 3D-printed cardiac model, catheter tracking through electromagnetic sensors, and a mixed-reality interface to display the real-time positions of devices. This hybrid simulator is used for procedures such as TSP, optimizing clinician training by improving the understanding of spatial relationships and enabling independent repetitions in complex anatomical scenarios. Additionally, it can be used for pre-procedural planning. [36] introduced mixed reality (MxR) software to plan LAAO. Using CT images, the system creates 3D holographic anatomical models, allowing precise morphological analyses to be completed in under 10 minutes. The results suggest that MxR can enhance planning efficiency and accuracy, especially in complex anatomies [37] developed a mixed reality system that integrates [38] with real-time visualization on the HoloLens. The technology combines accelerated reconstruction with intuitive interactivity, enabling real-time adjustments during complex procedures such as cardiac ablations. Validated on volunteers, the system demonstrated high precision and minimal latency, highlighting its clinical potential. Liu et al. [39] created an AR system that utilizes CT and fluoroscopy to guide transcatheter interventions such as TSPs. The combination of QR-code tracking with holographic rendering offers an average registration accuracy of 0.42 mm, facilitating intraoperative planning and reducing the learning curve.

[40] explored using mixed reality headsets (MR-HMD) in cardiac catheterization. The system integrated fluoroscopy, vascular ultrasound, and hemodynamic data directly into the operator's view, eliminating multiple monitors. Results showed significant reductions in procedure times and increased operational efficiency without compromising complication rates. [41] evaluated the MantUS™ system, which combines ultrasound with real-time needle tracking in an MxR interface. Applied to vascular access models, it demonstrated improvements in access quality and reduced procedure times, especially in challenging anatomies such as the right femoral vein. MantUS™ proved to be a highly promising tool with the potential for further developments and clinical studies.

[42] discussed the use of mixed reality in TAVI procedures, integrating ultrasound and CT with real-time holographic visualizations. The technology enhances procedure ergonomics and efficiency, reducing reliance on external monitors and improving precision. [43] introduced a HoloLens 2 system to guide femoral arterial punctures in interventional cardiology. The system was found to be accessible and cost-effective, improving control over typical errors and reducing operating times. Physicians highlighted its utility for both interventions and e-learning.

XR technologies are transforming interventional cardiology by integrating multimodal data into interactive and dynamic environments. The studies demonstrate the potential to reduce procedural complexity, improve accuracy, optimize clinical training, and open new possibilities for real-time planning and execution. Future studies should explore advanced integrations, such as machine learning and dynamic simulations, to further advance the application of these technologies.

### Artificial Intelligence

This section reviews recent applications of artificial intelligence and deep learning techniques in image-guided cardiac interventions, focusing on their role in enhancing image processing, device tracking, navigation accuracy, and procedural support.

Table 5 summarizes the work related to the application of AI strategies in cardiac interventions, outlining the key aspects of the respective studies. It is evident that one of the primary areas where AI has a significant impact is in PCI and TCI, as elucidated by Nisar et al. (2022) [44]. They propose an alternative to catheter navigation relying on fluoroscopy, introducing a vascular navigation system guided by ultrasound. The described solution is grounded in deep learning, specifically utilizing a U-Net network architecture. This architecture, based on ultrasound images US (ICE) of the vessels, reconstructs the vascular route, enabling navigation by a guidewire or tracked catheter. The network's output was compared to ground truth through manual segmentations, validated by experts, resulting in an accuracy of 90%, as indicated by the Dice coefficient. Typically, PCIs involve image guidance through X-ray angiograms, where coronary arteries are visualized using X-ray opaque contrast agents. Interventional cardiologists often navigate instruments using noncontract fluoroscopic images due to the potential risk of kidney failure associated with increased contrast agent usage. When relying on fluoroscopic images, interventional cardiologists must mentally reconstruct anatomical details.

**Table 5:** Summary of representative review works related to the application of AI methods

Field	Study	Modality	Goal	Method	Population	Comments/Main Conclusion
AI in PCI	[64]	X-Ray	Patient-specific DCR using angiographic images only.	U-Net.	34 PCI cases.	Accurate motion compensation without ECG.
AI in TCI	[44]	US (ICE)	DL-based vascular navigation with ICE images.	U-Net.	88 original images.	Achieved 90% Dice; ready for navigation system.
AI in Catheter Tracking (PCI)	[18])	X-Ray	Real-time DCR to reduce contrast use.	Bayesian filtering method and CNN.	N/A	Real-time capable; clinically integrable.
	[65]	X-Ray	Landmark and catheter tip detection.	CAM.	N/A	Outperforms baseline in X-ray angiography.
	[51]	X-Ray	Landmark detection in 3D-printed hearts.	2-stage CNN	N/A	Promising for broader cardiac applications.
	[66]	X-Ray	Real-time catheter balloon detection.	U-Net	N/A	Ready for online integration.
	[45]	MRI	Real-time catheter balloon detection.	U-Net.	12 patients.	Ready for online integration.
	[17]	US	3D catheter localization via segmentation.	U-Net 3D.	25 images.	Error smaller than catheter diameter.
	[67]	US	3D catheter shape estimation.	U-Net 3D and Kalman Filter.	N/A.	Robust to noise and complexity.
	[68]	US	Catheter detection in echo.	CNN in first phase.	N/A.	Fast and robust detection.
AI in Catheter Tracking/ Reconstruction (PCI)	[46]	X-Ray & US	Register 2D X-ray with 3D US.	U-Net.	N/A	Coarse 3D catheter reconstruction.
AI in Catheter Segmentation	[47]	US	3D segmentation in Frustum images.	CNN.	N/A	0.67 Dice in 3 s; real-time viable.
	[48]	US	Weakly supervised 3D segmentation.	CNN.	70 US volumes.	Validated with bounding-box labels.
AI in Image Fusion	[49]	X-Ray	Motion compensation for catheterization.	CNN.	64 sequences.	Successfully integrated in real application.
AI for X-ray Image Denoising	[50]	X-Ray	DL-based X-ray denoising.	UDDN in CNN	N/A	Better PSNR/SNR than previous methods.

*CAM* cascade attention machine, *DCR* dynamic coronary road mapping

In this context, [18], propose a novel dynamic approach to coronary mapping to enhance visual feedback and reduce contrast usage during PCI. They employ a method comprising a Bayesian filter and a convolutional neural network (CNN), compensating for cardiac and respiratory vessel movements induced by Electrocardiogram (ECG) alignment and catheter tip tracking in X-ray fluoroscopy. On the other hand, alternative approaches are suggested by authors like [45], who advocate MRI as a promising substitute for X-ray fluoroscopy in guiding CCP. MRI allows for soft tissue visualization, avoids ionizing radiation, and provides superior hemodynamic data. Presently, MRI-guided procedures necessitate frequent manual tracking of the imaging plane during navigation to follow the tip of a gadolinium-filled balloon catheter, leading to unnecessary procedural prolongation and complexity. Consequently, they developed an automatic, parameter-free, deep-learning-based post-processing pipeline (utilizing the U-Net architecture) for real-time catheter balloon detection. The system exhibited an overall accuracy of 98.4 ± 2.0%, with a deep learning-based segmentation step taking approximately 10 ms per image, indicating compatibility with real-time constraints. 3D ultrasound imaging has emerged as an appealing choice for guiding interventions, offering the potential to enhance the precision and efficiency of cardiac procedures. Effective catheter localization in 3D cardiac ultrasound (US) is crucial for optimizing outcomes. [17], present a catheter localization method for 3D cardiac US utilizing patch-wise semantic segmentation with model tuning. They employ a U-Net 3D network architecture trained with cross-entropy focal loss, emphasizing samples that pose classification challenges. Additionally, a dense sampling strategy is adopted to address the highly unbalanced catheter occupancy in 3D US data. Rigorous experiments conducted on a challenging ex-vivo dataset demonstrate that the proposed method achieves a catheter segmentation F-1 score of 65.1%, surpassing state-of-the-art approaches. As a result, the method can locate RF ablation catheters with an average error of 1.28 mm showcasing its potential for accurate and reliable catheter localization in 3D cardiac ultrasound applications. Still related to the location of the catheter, the authors [46, 47] and [48] proposes methods for catheter reconstruction in 3D US volumes and catheter segmentation, using deep CNNs methods.

[49], sought to enhance X-ray fluoroscopy with 3D anatomical overlays, a crucial technique for refining catheterization procedure guidance. However, cardiac, and respiratory movements often compromise the effectiveness of augmented fluoroscopy. To address this, the researchers explored motion compensation methods to update the overlay of a static model in response to respiratory and cardiac motion. They investigated the feasibility of motion detection between two fluoroscopic frames using a CNN. The integration of this CNN into the 3D-XGuide open-source software framework was demonstrated, expanding its capabilities to include automatic motion detection and compensation. The CNN was trained on reference data generated from rapid stimulation catheter tip tracking using template matching with normalized CC. The resulting CNN motion compensation model was encapsulated into an independent web service, enabling standalone use through a REST API. The model's performance was evaluated using 1,690 fluoroscopic image pairs from ten clinical datasets. The study introduces a novel CNN model-based method for automatic motion compensation during the fusion of 3D anatomical models with X-ray fluoroscopy. The successful integration with existing software applications showcases the potential of automatic motion extraction from 2D X-ray images using a CNN, representing a substantial advancement for reliable augmentation in catheter interventions.

The ongoing reliance on fluoroscopic X-ray imaging in cardiac catheter-based procedures, while beneficial, is associated with an escalation in radiation exposure for both patients and physicians. The imperative to reduce radiation dose, however, results in heightened noise and artifacts within the images, diminishing the clarity of discernible information. Consequently, there is a pressing need for advanced denoising methods tailored for low-dose X-ray imaging to enhance safety and reliability. In addressing this challenge, Luo et al. (*(PDF) *[50], propose the incorporation of UDDN (Ultra-dense denoising network) within the CNN framework for X-ray image denoising in cardiac catheter-based procedures. The introduced UDDN demonstrates commendable performance in both simulated and clinical cases, surpassing previous CNN architectures by achieving higher Peak Signal-to-Noise Ratio (PSNR) and Signal-to-Noise Ratio (SNR). This advancement signifies a notable stride towards mitigating noise-related challenges and enhancing the overall quality of X-ray images in cardiac interventions.

**Fig. 8:**
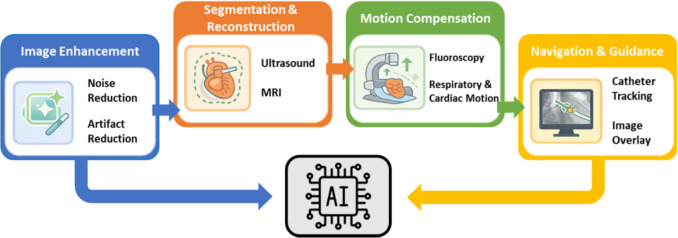
Conceptual overview of the transversal role of artificial intelligence within image-based cardiac navigation workflows. AI-based methods support multiple stages of the pipeline, including image enhancement, segmentation and reconstruction, motion compensation, and navigation and guidance, highlighting their integrative rather than isolated contribution to catheter-based interventions

Figure 8 illustrates the transversal role of deep learning within image-guided cardiac interventions, showing how AI-based methods are applied across different stages of the navigation pipeline, including image enhancement, segmentation and reconstruction, motion compensation, and procedural guidance. These studies collectively highlight the transversal role of AI across multiple stages of the image-based navigation pipeline, reinforcing its integration with imaging, modeling, and procedural workflows rather than its treatment as an isolated component.

## Discussion

With a growing geriatric population expected to triple by 2050, minimally invasive image-guided procedures are becoming increasingly popular and necessary for treating a variety of diseases [51]. Structural heart diseases are typically addressed through minimally invasive interventions, particularly via catheter. Multimodal registration of cardiac images plays a fundamental role in these procedures, providing complementary information for the fusion of cardiological images and facilitating cardiac image segmentation. This enables a more comprehensive analysis of cardiac functions and pathologies, with numerous clinical applications, such as monitoring disease progression, quantifying the effectiveness of treatment mechanisms, surgical planning, and intra-operative navigation.

Echocardiography, CT, MRI, and fluoroscopy are not only essential for diagnosing SHD, but they also play a pivotal role in real-time navigation during catheter-based interventions. Intraoperative echocardiography, especially TEE and ICE, allows real-time visualization of cardiac structures, guiding catheter placement with precision. For instance, during ASDC or LAAO, ICE provides real-time feedback on the spatial relationship between the catheter and target structures, reducing the need for fluoroscopy and minimizing radiation exposure. Fluoroscopy, often combined with contrast agents, is still heavily used for visualizing catheters in dynamic motion during interventions, such as TAVI. However, to enhance soft tissue visualization, hybrid imaging techniques, like the fusion of echocardiography and fluoroscopy, are increasingly used to overlay anatomical details from ultrasound onto fluoroscopic images, improving catheter guidance and device positioning.

CT and MRI, while typically used for pre-procedural planning, are also integral to navigation when combined with real-time imaging modalities. For example, 3D reconstructions from CT or MRI scans are often used to create patient-specific models of cardiac structures, providing a roadmap for the procedure. These 3D models are then integrated with real-time fluoroscopic or US images during the intervention, offering enhanced anatomical detail and aiding in the precise placement of catheters and devices. MRI, though less frequently used intraoperatively due to technical constraints, provides unparalleled soft tissue contrast and is valuable in evaluating cardiac function and tissue viability, guiding catheter movements in highly complex procedures like myocardial ablations. X-ray and fluoroscopy, despite their limitations regarding soft tissue contrast, remain key in tracking catheter positions throughout the procedure, while minimizing radiation exposure through enhanced imaging protocols. The integration of these modalities enables clinicians to navigate complex anatomy in real time, ensuring safer and more effective interventions.

The advantages of 3D US in guiding percutaneous procedures have already been highlighted. This modality also enables IF, where echocardiographic and fluoroscopic images are synchronized and displayed from the same visual perspective, providing a better spatial relationship of intracardiac anatomy. The position and orientation of the TEE probe are co-registered with the fluoroscopic image, with markers applied to important structures in the 3D images, which are then simultaneously shown in fluoroscopy, thus assisting in interventions. This type of FI addresses some issues associated with fluoroscopy, such as poor visualization of soft tissues and the use of only 2D monitors, although it remains a contrast-free and radiation-free technique. Currently, the most common applications of this technology include TAVR deployment, especially in patients with non-calcified valves and paravalvular leakage, ASDC, and LAAC. The feasibility and spatial accuracy of pre-procedural 3D CT and MRI images for 3D rotational fluoroscopy registration to guide interventional procedures in patients with congenital heart disease have also been investigated and are referenced in this document. The studies we researched did not provide many details about fusion schemes, and most of them achieved static fusion. There were only a few commercially available software products for real-time fusion between dynamic images. For example, EchoNavigator is a software tool that allows real-time synchronization and fusion of 2D, and 3D TEE with fluoroscopy images. The coordinate system of the two imaging modalities can be aligned based on the location and tracking of the TEE probe. This software can facilitate complex percutaneous procedures and reduce procedure duration as well as radiation dose.

The traditional process of cardiac image registration is essentially an iterative optimization procedure, which can be quite time-consuming, especially when dealing with non-rigid images. On the other hand, DL-based registration methods have demonstrated computational efficiency, as evidenced by studies such as Nisar et al. (*(PDF) Towards Ultrasound-Based Navigation: Deep Learning Based IVC Lumen Segmentation from Intracardiac Echocardiography*, n.d.). These methods have succeeded when applied to multimodal cardiac images, as shown in study conducted by [46]. In general, these methods require extensive anatomical labelling for supervised training in neural networks (H. [18]). Additionally, the concepts of group registration and combined computation have recently emerged and have been employed in multimodal registration and segmentation of cardiac images [49]. Compared to pair-wise registration, group registration can simultaneously and impartially handle various image modalities. Therefore, the introduction of group learning can make DL-based multimodal image registration even more efficient. However, due to the wide variety of modalities and clinical scenarios in cardiology, there are still no generalized automatic methods for this task.

3D printing is used in patients with congenital heart disease to accurately visualize complex anatomy and represent intricate intracardiac relationships. Most cardiac 3D printing relies on contrast-enhanced X-ray, CT, and MRI datasets, producing high-resolution images of most intracardiac structures [33]. Recently, there's a growing use of 3D models derived from echocardiographic data, providing high accuracy, though with technical limitations due to lower spatial resolution, point artifacts, and reduced signal-to-noise ratio compared to other imaging modalities. 3D-printed models are becoming crucial in treating patients with coronary disease. Despite gaining prominence, producing 3D models involves segmenting image data before printing, which is time-consuming and introduces a potential source of human error. In this context, accurate anatomical segmentation represents a critical first step in the cardiac 3D printing workflow and is often a major bottleneck due to its reliance on image quality, imaging modality, and operator-dependent manual or semi-automatic procedures. Most current 3D printing pipelines are based on CT or MRI data, while echocardiography-derived models may suffer from lower spatial resolution and image artifacts, further increasing segmentation complexity. Recent advances in artificial intelligence, particularly deep learning–based segmentation methods [46]. have demonstrated strong potential to reduce manual effort, improve reproducibility, and facilitate the integration of multimodal imaging data into patient-specific anatomical models. The incorporation of AI-driven segmentation within 3D model generation pipelines is therefore expected to enhance the reliability, efficiency, and practical applicability of cardiac 3D printed models for procedural planning and image-guided navigation. Additionally, resulting models are static and rarely encompass all valve structures, and analysing the model may require cutting it. 3D printing is valuable in determining the most appropriate surgical approach for specific heart lesions. It can also be a crucial tool in planning extracardiac surgeries in patients with congenital heart disease, such as high-risk antilocalization surgery in cases of pulmonary atresia and large aortopulmonary collateral arteries. Three-dimensional models can provide detailed information about patients' airways. Currently, no imaging modality can demonstrate all necessary anatomical features in complex lesions to guide surgical planning. Hybrid 3D printed models, combining data from different imaging modalities, have the potential to provide superior diagnostic information to individual modalities, aiding in practical simulations of the ideal surgical approach in challenging cases [32]. Combining the strengths of different imaging techniques is now feasible, allowing the printing of hybrid 3D models for heart lesions.

XR environments can offer 3D representations of cardiac anatomy and provide an interactive platform, allowing surgeons or interventionists to manipulate and rotate images according to their perspective in all directions. Additionally, XR can overcome the drawbacks of other 3D technologies, capable of representing dynamic structures like valves and their supporting apparatus and avoiding the additional time and cost of creating 3D printed physical models. Interrogation of images using slice planes and measurement of cardiac structures is also possible in the XR environment. XR holds significant potential for planning surgical and catheterization procedures in congenital heart diseases, where understanding the spatial relationships of structures is crucial [35]. Advances in lightweight and transparent HMDs and manual gesture control have made 3D intra-procedural guidance possible.

Compared to existing reviews, which often focus on specific aspects such as 3D printing, XR, or the application of multimodal imaging techniques in particular clinical contexts, this review stands out for its comprehensive and integrative approach in analysing a broad range of methodologies used in image-based medical navigation systems for catheter-based cardiac procedures. While previous reviews, such as those by [52] [53], explore specific technologies or the application of individual imaging modalities, our review differentiates itself by synthesizing the design and development decisions of research teams working on the creation of innovative and multifaceted solutions. Furthermore, by categorizing the methodologies into six major groups (Image Enhancement and Tracking, Image Fusion and Reconstruction, 3D Modelling/Printing, Extended Reality, and AI), we provide a more structured and comprehensive view that highlights how the combination of techniques can overcome challenges such as intermodality misalignment and multimodal data integration. In particular, in the context of catheter-based cardiac interventions, current clinical practice indicates that progress in image fusion and multimodal imaging integration has a more immediate impact on procedural guidance than fully robotic solutions, which remain limited in routine clinical adoption. This observation further supports the proposed structure of the review, which prioritizes image-based navigation workflows that are directly aligned with contemporary interventional practice. This differentiated perspective offers a critical view of current navigation strategies and points to future development directions that can have a direct impact on clinical practice, enhancing the utility and effectiveness of these systems in interventional cardiology.

## General Current State of Research and Perspectives

The last five years have witnessed a remarkable evolution in image-based navigation systems for structural heart disease interventions. The studies analyzed in this review demonstrate a growing sophistication in how imaging is used, not just to visualize, but to guide, personalize, and optimize complex catheter-based procedures. The integration of multimodal data has laid the foundation for high precision interventions, but the true future of this field lies in intelligent and interoperable systems.

One of the most transformative drivers in this space is artificial intelligence. Across the reviewed categories, image enhancement, tracking, fusion, 3D modelling, and extended reality, AI plays a pivotal role in overcoming current limitations. It enables automatic segmentation of cardiac structures, enhances real time image fusion through deep registration algorithms, and improves navigation by predicting catheter trajectories or detecting key anatomical landmarks. These capabilities reduce operator variability, shorten procedure times, and boost procedural safety.

Extended reality technologies have been explored in cardiac interventions for more than a decade, initially as experimental visualization tools primarily aimed at education, pre-procedural planning, and anatomical understanding. Around 2019, XR applications in cardiology were still largely limited by hardware constraints, limited integration with real-time imaging, and challenges related to accuracy, ergonomics, and clinical workflow adoption. Over the past five years, however, advances in head-mounted displays, spatial tracking, visualization software, and multimodal image integration have progressively increased the feasibility of XR-based guidance, particularly for procedural planning, training, and selected intra-procedural support scenarios. It is worth noting that some of the most advanced XR implementations in surgery have been integrated with robotic-assisted systems, which enable precise instrument tracking and controlled execution. However, such robotic platforms are not yet widely adopted in catheter-based cardiac interventions, where navigation remains predominantly physician-driven and imaging-centered. For this reason, robotic-assisted solutions were considered outside the scope of this review, while XR is primarily discussed as an imaging and visualization technology that complements, rather than replaces, conventional image-guided workflows. While widespread adoption in real-time cardiac navigation remains limited, ongoing improvements in device usability, tracking accuracy, and integration with imaging and AI-driven workflows suggest that XR is likely to play an increasingly important role in future image-based navigation systems.

At the same time, the integration of artificial intelligence with 3D printing and extended reality environments opens new doors for procedural planning and simulation. Patient specific phantoms, augmented by real imaging data and computational models, are now being used for rehearsal, device testing, and even intra procedural guidance when coupled with virtual overlays. This synergy between physical and digital imaging technologies brings us closer to a fully immersive, predictive, and personalized intervention workflow.

However, significant challenges remain, particularly in ensuring the synchronization and alignment of multimodal data in real time. The combination of modalities with different spatial and temporal resolutions, such as CT, MRI, ICE, and fluoroscopy, demands robust registration algorithms and standardized protocols for data interoperability. Moreover, ensuring real time performance in artificial intelligence powered systems, without compromising accuracy, remains an open technical frontier.

Looking ahead, the most promising perspective is the convergence of imaging, artificial intelligence, and procedural intelligence. In this context, procedural intelligence and automated workflow analysis are gaining increasing attention as key enablers of intelligent navigation systems. By identifying procedural phases, tool usage patterns, and workflow transitions in real time, these approaches can provide context-aware guidance, automate routine tasks, and support decision-making during complex interventions. When combined with imaging and artificial intelligence, workflow-aware systems have the potential to improve procedural efficiency, reduce operator variability, and enhance patient safety, representing an important step toward fully integrated and adaptive image-based navigation platforms. Navigation systems are no longer isolated tools; they evolve into integrated platforms where information flows seamlessly between acquisition, interpretation, planning, and guidance. This interconnected approach will be critical for managing the growing complexity of structural heart interventions and ensuring optimal outcomes across diverse clinical settings.

## Conclusion

Imaging exams play an essential role in clinical decision-making in SHD, being used before, during and after procedures to assess suitability, feasibility, and follow-up. This review highlights recent advances in multimodal cardiac imaging techniques as a surgical navigation system. Several cutting-edge technologies, including tracking, image fusion, extended reality, 3D printing and AI, were covered. It is concluded that, although the development of ML, particularly DL, has significantly advanced multimodal analysis of cardiac images, important challenges remain for its effective use in image-guided navigation. These include the need for robust and generalizable models across imaging modalities, real-time performance under clinical constraints, reliable integration with multimodal image fusion pipelines, and reduced dependency on extensive manual annotations. Emerging solutions highlighted in this review include AI-driven automated segmentation, learning-based multimodal registration, workflow-aware guidance systems, and the tighter integration of machine learning with real-time imaging and navigation platforms. Addressing these challenges will be essential for translating machine learning based methods into reliable clinical image guidance systems.

## Data Availability

No new data were generated or analyzed in this study.

## Declarations

### Authors and Editorial Board Members

Nothing to report.
